# Insight into Thermal Stress Distribution and Required Reinforcement Reducing Early-Age Cracking in Mass Foundation Slabs

**DOI:** 10.3390/ma14030477

**Published:** 2021-01-20

**Authors:** Barbara Klemczak, Aneta Żmij

**Affiliations:** Faculty of Civil Engineering, Silesian University of Technology, Akademicka 5, 44-100 Gliwice, Poland; aneta.zmij@polsl.pl

**Keywords:** hydration temperature, thermal stress, early-age cracking, foundation slabs, reinforcement

## Abstract

The heat released during cement hydration results in temperature-induced non-uniform volume changes in concrete structures. As a consequence, tensile thermal stresses of significant values may occur. The level of these stresses can be lowered by using various technological measures during the construction process and a proper concrete mix composition. Nevertheless, the application of an appropriate reinforcement is a reliable method for controlling the width and spacing of possible cracks. The rules for calculating this reinforcement are not precisely detailed in the standards devoted to concrete structures. Additionally, the correct calculation of the reinforcement requires the identification of the tensile stress distribution in a mass slab. The presented study provides insight into stress distribution and relevant reinforcement for controlling early-age cracks of thermal origin. The existing standards and guidelines are discussed and clarified. The possible paths for calculating the reinforcement are proposed through the example of mass foundation slabs with different levels of external restraints. The results indicate a significant impact of the calculation method as well as the restraint conditions of the slab on the area of required reinforcement.

## 1. Introduction

Foundation slabs are exposed at an early age to elevated temperatures resulting from the heat released during cement hydration. These thermal loads coming from the material itself can induce a significant level of stress. Primarily, self-induced stresses result from the nonlinear temperature and strain distribution at the slab cross-section. Furthermore, the external limitations of thermal deformations induce restrained stresses. In slabs with a considerable thickness, especially foundation slabs, the latter type of stress typically is of a lower magnitude and less concern. The mentioned stresses often exceed the tensile strength of young concrete that is just developing mechanical properties. Although cracks in reinforced concrete structures are acceptable, their width is limited due to serviceability reasons. Moreover, early cracks require special attention, as they may expand during the later life of the structure under the impact of mechanical loads. Additionally, the crack width is limited to protect reinforcement against corrosion hazards. The level of induced stresses and the cracking risk depend on many factors, which have recently been categorized into five basic groups [1].

The first group is related to the details of the slab resulting from the load-bearing analysis due to the mechanical loads. The volume and the thickness of the slab, the external restraining conditions, and the details of the reinforcement fall into this category. It is generally believed that the thermal gradients may cause cracking in structures with a large volume of embedded concrete, generally described as mass concrete [2,3,4]. This is justified, because in members of considerable thickness, the self-heating of concrete, resulting from the cement hydration process, can reach tens of degrees Celsius. However, the term “mass concrete” is imprecise [2]. An attempt to clarify this matter is represented by classification based on the surface modulus, defined as the ratio between the area of surfaces subjected to environmental cooling and the total volume of the structure [5]. Considering the surface modulus, the structure is classified as a mass concrete if its value is lower than 2 m^−1^. Then, the expected self-heating of concrete may exceed 20 °C, and the structure is recognized as sensitive to early-age cracking. Following this definition, foundation slabs with a thickness greater than 1m can be considered a mass structure [5].

The next four groups correspond to the concrete mix composition, the specificity of the construction process, the environmental conditions, and the external loading conditions. Among these factors, the composition of the concrete mix is of particular concern, since the hydration heat substantially depends on the amount and type of cement used [6,7,8,9].

Early-age thermal effects in concrete structures have been the subject of extensive research since the thirties of the twentieth century, when the construction of large dams raised problems with hydration heat. Some studies are devoted to hydration heat [10,11,12,13], temperature and stress variation in concrete members [14,15,16,17,18], the analysis of cracking risk [19,20,21,22], as well as measures preventing this risk [23,24,25,26]. Thus, the behavior of early-age concrete concerning these issues is fairly well recognized. Not many articles have been devoted to the role of reinforcement and the methods for its calculation in mass concrete members under thermal loading. Admittedly, reinforcement does not limit the level of the load and thermal stresses. Consequently, reinforcement does not prevent cracking but is rather for limiting the width and spacing of the cracks to acceptable values. The available works discuss mainly early-age cracking in reinforced concrete walls on slab [27,28] or general requirements for shrinkage and temperature reinforcement in concrete structures [29,30]. Issues concerning early thermal cracks and minimum reinforcement in mass foundation slabs are outlined in [31,32], but not all aspects of the discussed complex issue are covered.

In Europe, the determination of reinforcement for cracking control is based on the design recommendations provided by Eurocode 2 [33], which is actually applicable for typical mechanical loads and does not accurately specify the early-age thermal behavior of concrete members. The topic is more widely discussed in the CIRIA C660 [34] and CIRIA C766 [35] guidelines, described as the British commentary on the Eurocode standards. Nevertheless, the recommendations and worked examples focus on ground slabs without external restraints. Furthermore, the distribution of stresses throughout the concrete hardening period is not precisely analyzed, and especially the cooling phase is neglected. Other national standards can be characterized in a similar way because they usually provide only very general recommendations for reinforcement limiting cracks of thermal origin [36,37].

Therefore, despite significant research in the field of early-age thermal effects, there is no systematic approach devoted to the calculation of reinforcement considering the development of strains in mass foundation slabs with different restraint conditions. The methods for calculating the reinforcement in massive foundation slabs are also not thoroughly described in the available standards. In the authors’ opinion, the calculation of the appropriate reinforcement requires understanding the distribution of thermal stresses resulting from the hydration temperature. Hence, the article discusses the distribution of thermal strains as well as indicating the areas of tensile strains in the heating and cooling phase of the mass foundation slab. Therefore, the different behavior of the foundation slab with a slip layer and the slab with limited deformation freedom was demonstrated. Next, guideline recommendations that can be used to calculate the necessary reinforcement and their inaccuracies and discrepancies are presented.

Compared to other works, the novelty of the article is related to the analysis of mass foundation slabs with different degrees of external restraint as well as discussing possible methods for calculating the reinforcement. The original method for the assessment of tensile strain of thermal origin is also presented in Section 4. The study also draws attention to tensile stresses of significant values, which may arise in slabs with limited deformation freedom during the cooling phase. This issue has not been sufficiently highlighted in the literature so far. The presented calculation example and the comparison of the required reinforcement determined based on the different methods can be useful for practicing engineers, who indicate problems in determining the appropriate level of reinforcement in mass foundation slabs. 

## 2. Problem Overview

**Figure 1:**
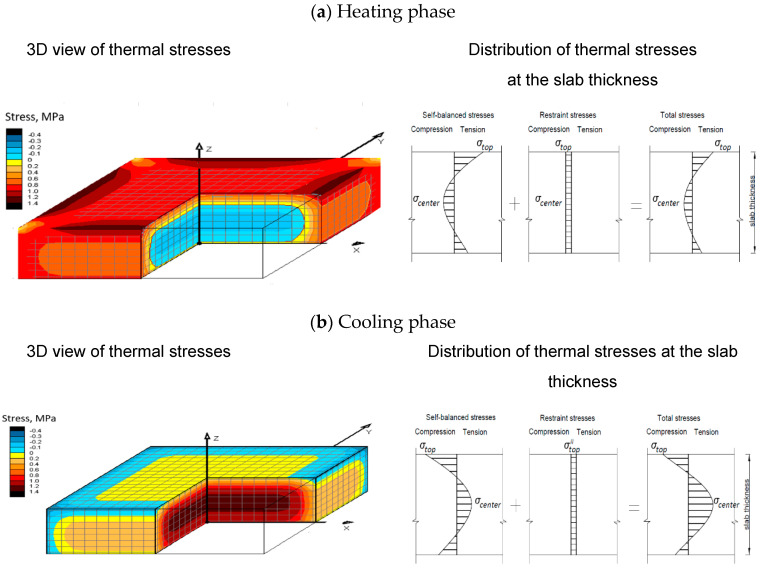
Distribution of thermal stresses in a foundation slab with a considerable thickness: (**a**) heating phase, (**b**) cooling phase.

Generally, thermal stresses arise when concrete structure dilation due to thermal loads is restrained. Two types of restraints can be distinguished in mass foundation slabs. Internal restraints result from thermal gradients and non-uniform volume changes at the cross-section of the slab. Thus, self-induced stresses are induced. Consequently, the different volume changes of the core and surfaces induce both tensile and compressive stresses along the slab width. In the heating phase, the surfaces are restrained by the inner parts of the slab, and tension is observed in the subsurface regions (Figure 1a). In the cooling phase, stress inversion occurs and compression is observed at the slab surfaces, while tensile stresses appear in the interior (Figure 1b).

Additionally, the existing external restraints limit the free thermal expansion and contraction of the slab during concrete curing. In foundation slabs, such restraint exists along the contact surface of the slab and subbase. The limitation of deformation freedom can also be caused by an additional restraining element such as piles. The character of the restrained thermal stresses is different from that of self-induced stresses. In the phase of temperature increase, restrained thermal stresses of a compressive nature develop in the whole volume of the slab (Figure 1a). After reaching the maximum temperature, the cooling phase starts, and tensile restrained stresses are observed (Figure 1b). The level of restrained stresses depends on the degree of restraint existing between the slab and the restraining element [38]. Generally, based on experience and numerical studies, restrained stresses are recognized to be of lower importance in ground slabs with considerable thickness [38]. Similarly, the shrinkage strains associated with concrete curing gain negligible value compared to thermal deformation [39].

Following the described development of thermal stress, cracks can be induced in the heating phase at the surface of the slab, where the tensile stresses exist. These cracks may occur within the first few days after concrete placement, when the tensile strength of concrete is relatively low. Later, in the cooling phase, cracks can arise in the inner part of the slab, which was previously compressed and afterward is subjected to tension.

## 3. Existing Recommendations for Reinforcement Calculation

### 3.1. General Remarks

Mass concrete structures may be insufficiently reinforced for the discussed thermal stresses and are thus prone to cracks of considerable width. Many examples of early-age cracking due to insufficient reinforcement can be found in the thematic literature [5,40,41]. Undoubtedly, apart from providing suitable reinforcement limiting the early-age crack width, thermal stresses can be minimized by the proper technological measures. Nevertheless, the use of the appropriate reinforcement in the discussed structures remains a primary method for the effective limitation of the thermal crack width.

Cracking in concrete starts when the induced tensile strain exceeds the maximum strain that concrete can withstand without crack formation—namely, the ultimate tensile strain capacity. The value of the ultimate tensile strain capacity, *ε_ctu_*, depending on the concrete class and the aggregate type, does not surpass 100 *με*, for early-age concrete [35]. Therefore, assuming the coefficient of thermal expansion to be equal to °*με*/°C, the maximum permissible temperature difference is Δ*T* = 10 °C.

As the determination of the actual thermal gradients caused by cement hydration heat brings many troubles, a simplified method can be used for the reinforcement calculation. The method assumes that in an element with tensile stresses of thermal origin, the minimal area of reinforcement, *A_s,min_*, should be provided, considering the limit values of the stresses causing cracking in the concrete. Thus, the actual level of induced thermal stresses is ignored. According to Eurocode 2 [33], the required limit of the crack width is satisfied by using the suggested bar size and spacing. Undoubtedly, the advantage of this approach is its relatively simple calculations, which do not require a significant amount of work—there is no need to analyze the material and technological factors related to the casting process of the slab. This approach is also safe since, regardless of the magnitude of the actual thermal stress, the assumed maximum crack width is not exceeded. The main disadvantage of the solution may be the oversizing of the reinforcement, since the induced tensile stresses can be lower than the tensile strength of concrete, especially under favorable technological conditions. 

On the contrary, a more accurate method based on the determination of the magnitude of thermal strains and stresses can be applied. It should be mentioned that the evaluation of the early-age hardening temperature and the resulting stresses is a complex task, since a large number of technological and material factors determine the size and the nature of volumetric changes. Therefore, the material (the amount and type of cement, type of aggregate) and technological data (i.e., casting technology, the variation in ambient temperature, the initial temperature of the concrete) must be considered. Furthermore, all the technological and material data assumed for the calculations must be retained during the construction of the slab, because their changes influence the required area of reinforcement. In conclusion, this approach, although described as more accurate, may carry the risk of underestimated reinforcement if the actual maturation conditions for the concrete are less favorable than assumed in the design. In the simplified method, regardless of the occurrence of less favorable conditions, the assumed crack width will be fulfilled.

### 3.2. Reinforcement Area and Location

Generally, the minimum area of reinforcement, *A_s,min_*, required to control cracking in tension areas may be calculated based on Equation (1) [33,34,35]:

(1) $$A_{s,min}=k_{c}kA_{ct}\frac{f_{ct,eff}}{\sigma_{s}},$$

where $A_{ct}$—is the area of the slab cross-section in tension; $\sigma_{s}$ is the absolute value of the maximum stress permitted in the reinforcement immediately after the formation of the crack; *f_ct,eff_* is the mean value of the tensile strength of the concrete effective at the time when the cracks may first be expected to occur; k is the coefficient considering the effect of non-uniform self-equilibrating stresses; *kc°* is *°* the coefficient considering the stress distribution within the section immediately before cracking.

Particular standards and guidelines differ in the methods of determining the values of *A_ct_*, *σ_s_*, *f_ct,eff_*, *k* and *k_c_*in Equation (1).

In detail, the Eurocode 2 standard [33] does not provide detailed recommendations for the application of Equation (1) for mass foundation slabs subjected to early-age thermal effects. First, no clarification regarding the area of the concrete in tension $A_{ct}$ and the distribution of induced thermal stresses is given. Next, the mean value of the concrete tensile strength, *f_ct,eff_ = f_ctm_(t)* is recommended to be assumed for the concrete age, *t*, when cracks are expected. Simultaneously, the concrete age, t, is not specified. These inaccuracies have been discussed in [42,43], and using the German standard DIN EN 1992-1-1/NA [44] is recommended. In this standard, *f_ct,eff_* is equal to 0.5*f_ctm_*. The same assumption has been made in [5].

Coefficient *k*, considering the effect of non-uniform self-equilibrating stresses, depends on the cross-section dimension and is equal to:

- 1.0 for webs with h ≤ 300 mm or flanges with widths less than 300 mm,
- 0.65 for webs with h ≥ 800 mm or flanges with widths greater than 800 mm;
- intermediate values may be interpolated.

**Figure 2:**
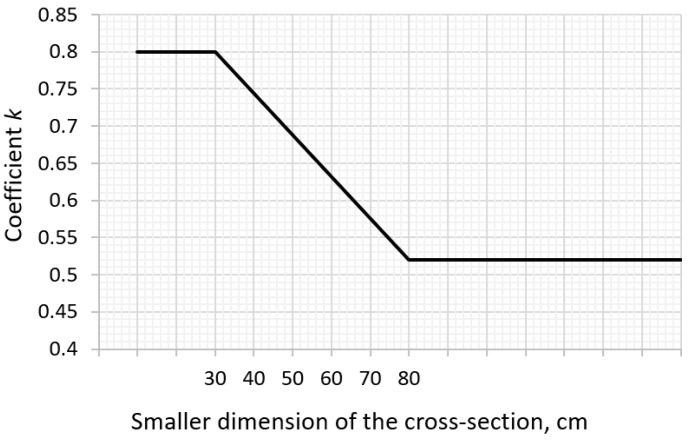
Coefficient *k* according to DIN EN 1992-1-1/NA [44].

Other shapes of cross-sections are not specified in the Eurocode 2 standard. The provisions of the German standard DIN EN 1992-1-1/NA [44] with the coefficient *k* depending on the smaller dimension of the element cross-section (Figure 2) are more general. Based on the German standard, it can be noticed that for a slab with a thickness greater than or equal to 80 cm, the discussed coefficient is smaller (*k* = 0.52) than that proposed by Eurocode 2 (*k* = 0.65).

Eurocode 2 recommends the value of the coefficient *k_c_* depending on the stress distribution in the cross-section at the moment preceding cracking, which is equal to 1.0 for pure tension. This conservative value is justified, since the existing self-equilibrating stresses are considered in the coefficient *k*.

Next, the maximum stress, *σ_s_*, allowable in the reinforcement immediately after the crack formation may be taken as the yield strength of the reinforcement, *f_yk_*. At the same time, the lower value of the maximum steel stress, depending on the applied bar diameter, is suggested to satisfy the crack width limits *w_lim_* (Table 1). The listed values are derived for the concrete class C30/37 and the concrete cover of 25 mm.

It is worth noting that the stress *σ_s_*, listed in Table 1, is based on Equation (2) given by Rüsch and Jungwirth [45], according to which ensuring the condition *w < w_lim_* requires the use of reinforcement with a diameter of $\phi_{s}$, satisfying the condition:

(2) $$\phi_{s}=\frac{3\;\tau_{1}\;w_{lim}\;E_{s}}{f_{yk}^{2}}$$

where *τ*_1_ id the concrete bond strength for horizontal bars taken as $\tau_{1}=0.15f_{cm}$; $E_{s}$ id the elastic modulus of steel.

Thus, for diameters *ϕ* other than diameter *ϕ_s_*, the stress *σ_s_* should be corrected using Equation (3):

(3) $$\sigma_{s,red}=f_{yk}\sqrt{\phi_{s}/\phi }$$

The British guidelines CIRIA C660 [34] and CIRIA C766 [35], complementary to Eurocode 2 [33], broadly describe early-age volume changes in concrete and give precise recommendations related to Equation (1). Therefore, the mean value of the concrete tensile strength, $f_{ct,eff}$, at the time when cracks are expected to develop, is taken as $f_{ct,eff}=f_{ctm}(t=3\;\mathrm{days}$). The proper values of the strength $f_{ct,eff}$ for concrete of various classes are given in Table 2. Furthermore, the cross-sectional area of the tensile zone $A_{ct}$ and the coefficients k and $k_{c}$ are based on the nature of the restraints (internal or external). The recommended values are summarized in Table 3.

Particular attention should be paid to the location of the reinforcement. In the heating phase, the tensile self-induced stresses arise in the surface zone and the reinforcement should be placed there. In CIRIA C660 [34] and CIRIA C766 [35], the tensile area is assumed to be a depth of 0.2 h (Table 3). This value results from the temperature profile at the cross-section, which can be approximated by a parabola. Furthermore, for the dominant internal restraint in the slab the stress and strain distributions have the same shape through the cross-section as the temperature profile. Consequently, the line separating tensile and compressive stresses is located exactly at 0.211 h due to the properties of the parabolic distribution.

German standard DIN EN 1992-1-1/NA [44] recommends using Equations (4)–(7) for the tensile area, calculated as:

(4) $$A_{sk}=h_{sk}\times 1m$$

(5) $$2h_{sk}=5a_{1}\;dla\;\;h\leq 5a_{1}$$

(6) $$2h_{sk}=4a_{1}+0,2h\;dla\;\;5a_{1}<h<30a_{1}$$

(7) $$2h_{sk}=10a_{1}\;dla\;\;h\geq 30a_{1}$$

where $a_{1}=c+0,5\emptyset$. Assuming the tensile depth to be equal to $h_{sk},$ the minimum area of reinforcement, $A_{s,min}$, is calculated based on Equation (8) [44]:

(8) $$A_{s,min}=A_{sk}\frac{f_{ct,eff}}{\sigma_{s}}$$

Considering the restrained stresses induced in the heating phase, it can be noticed that they are of a compression nature and thus do not require reinforcement.

**Figure 3:**
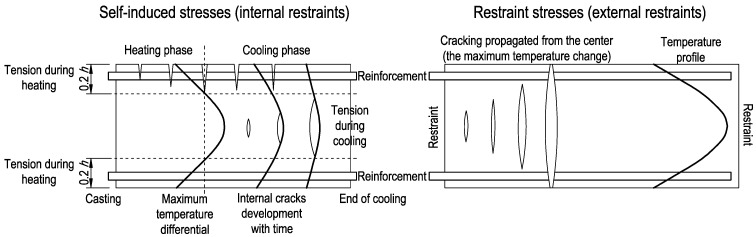
Cracking mechanism in mass slabs [34,35].

Generally, the cracking of the inner part of the slab due to tensile stresses occurring in the cooling phase is not considered in the discussed recommendations. Nevertheless, CIRIA C660 [34] and CIRIA C766 [35] mention the possibility of cracking inside the slab (Figure 3). The cracks can appear since the self-induced and restrained stresses add up in the cooling phase. Especially, the internal cracking may occur in elements of considerable thickness and with a high level of external restraint. The guidelines CIRIA C660 take this into account in the proposed increase in the *k*-factor to the value of 0.75 for sections with a thickness greater than 800 mm. This is a greater value in comparison to the recommendations of Eurocode 2 and CIRIA C766 (*k* = 0.65), as well as the recommendations of the German standard (*k* = 0.52). In this way, an increased near-surface reinforcement area can limit the width of surface cracks, which may be magnified due to internal cracks propagating from the center to the surface of the element. To sum up, the guidelines allow for the formation of cracks in the slab interior but limit their width in the surface zone by an increased amount of near-surface reinforcement. These assumptions also adjust the belief that the surface cracks arising in the heating phase may partially close in the cooling phase. Thus, the role of the surface reinforcement is also to limit the developing internal crack.

### 3.3. Cracking Width and Spacing

For the foundation slabs, which are subjected mainly to internal restraint or additional edge restraint, the crack width can be calculated using Equation (9) [33,34,35]:

(9) $$w=s_{r,max}\varepsilon_{cr}=\varepsilon_{cr}\left[3.4c+0.425\frac{k_{1}\phi }{\rho_{p,eff}}\right]$$

where $s_{r,max}$ is the maximum crack spacing, m; c is the concrete cover, m; $k_{1}$ is the coefficient taking into account the bond properties of the reinforcement (Eurocode 2 [33] recommends the value of 0.8 for high bond bars and 0.7 for typical bars, however [34,35] recommend the higher value $k_{1}=1.14$); ϕ is the bar diameter, m; $\rho_{p,eff}$ is the reinforcement ratio, calculated as $\rho_{p,eff}=A_{s}/A_{c,eff}$; $A_{s}$ is the reinforcement area, $m^{2}$; $A_{c,eff}$ is the effective area of concrete in tension around the reinforcement to a depth of $h_{c,eff}$.

It should be noted that the effective area of concrete in tension, $A_{c,eff}$, differs from the area of concrete in tension, $A_{ct},$ used for the calculation of the reinforcement area. While the area of concrete in tension, $A_{ct}$, depends on the form of restraint (Table 3), for the crack width calculation the effective area of concrete in tension $A_{c,eff}$ is taken regardless of the restraint conditions. Moreover, only the area around the reinforcement is considered, to a depth of $h_{c,eff}$, calculated from Equation (10) [33,34,35]:

(10) $$h_{c,eff}=min\left\{\frac{h}{2};\;2.5\left(c+\phi /2\right)\right\}$$

The crack inducing strain $\varepsilon_{cr}$ is calculated from Equation (11) [35]:

(11) $$\varepsilon_{cr}=\varepsilon_{r}-0.5\varepsilon_{ctu}$$

The tensile strain capacity, $\varepsilon_{ctu}$, depends on the type of aggregate applied in concrete [34,35]. Early-age crack-inducing strain, $\varepsilon_{r}$, is calculated using Equation (12):

(12) $$\varepsilon_{r}=K_{1}R\alpha_{T}\Delta T$$

where $K_{1}$ is the coefficient of stress relaxation due to creep under sustained loading, the recommended value is $K_{1}=0.65$ [34,35]; R is the restraint factor describing the degree of deformation freedom; $\alpha_{T}$ is the coefficient of thermal expansion, depending on the aggregate type [34,35], and if no data are available the value of 12 με/°C may be used; ΔT is the temperature difference.

## 4. Required Reinforcement Based on the Thermal Strain and Stress Analysis

The thermal stresses in foundation slabs can be evaluated using numerical or analytical methods. Undoubtedly, the numerical approaches provide a complex analysis of the thermal strains and stresses in the slab over the whole time of concrete hardening [7]. The analytical methods are considered as a simple evaluation of the induced stresses as well as more convenient for the engineering application [5,34,35].

Analytically, the thermal stress can be determined based on a restraint factor *R*, which defines the restraint stress from a fixation stress $\sigma_{fix}$, as shown in Equation (13):

(13) $$\sigma_{R}=R\sigma_{fix}$$

The stress that would occur in the fixed element can be calculated from Equation (14):

(14) $$\sigma_{fix}=\alpha_{T}\Delta TE_{cm,eff}$$

where $E_{cm,eff}$ is the effective modulus of elasticity of concrete with the consideration of creep effects.

Generally, the factor *R* represents the degree of restraint occurring in the structure. In the case of mass foundation slabs, its value depends on the type of induced stresses. For the internal restraints and the stress or strain at the top surface, the recommended value is 0.42 [34,35]. Considering the external restraints of mass foundation slabs, the existing recommendations are listed in Table 4. Due to the large length/thickness ratio, typical for the foundation slabs, the external restraint factor can be assumed as a constant value at the slab thickness.

**Figure 4:**
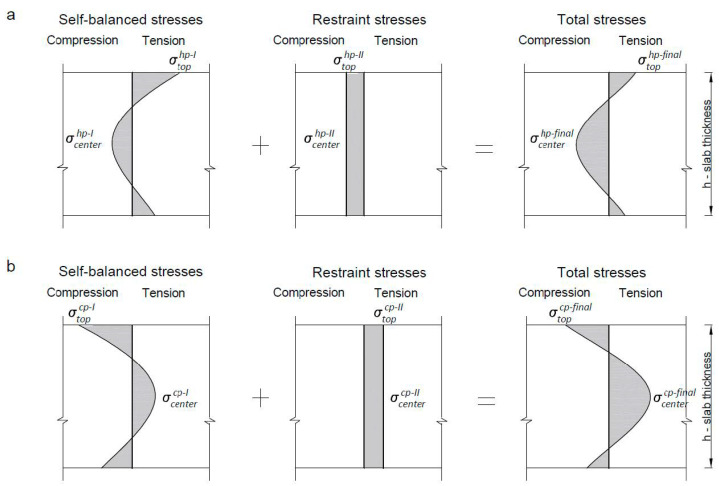
The distribution of self-balanced and restrained stresses: (**a**) heating phase, (**b**) cooling phase.

Assuming the parabolic distribution of the self-balanced strains and stress, Equations (15)–(22) can be used for their calculation. The maximum stresses in the heating phase (*hp*) can be determined (Figure 4) as follows:

(15) $$\sigma_{top,tension}^{hp-I}=0.42\alpha_{T}\Delta T_{1}E_{cm,eff}$$

(16) $$\sigma_{center,compression}^{hp-I}=-0.21\alpha_{T}\Delta T_{1}E_{cm,eff}$$

In the cooling phase (*cp*), the inversion of self-balanced stresses occurs:

(17) $$\sigma_{top,compression}^{cp-I}=-0.42\alpha_{T}\Delta T_{1}E_{cm,eff}$$

(18) $$\sigma_{center,tension}^{cp-I}=0.21\alpha_{T}\Delta T_{1}E_{cm,eff}$$

Restrained thermal stresses have a different character than self-balanced stresses. In the phase of temperature increase (heating phase), the whole slab is subjected to compression:

(19) $$\sigma_{top,compression}^{hp-II}=-R\alpha_{T}\Delta T_{2}E_{cm,eff}$$

(20) $$\sigma_{center,compression}^{hp-II}=-R\alpha_{T}\Delta T_{3}E_{cm,eff}$$

In the cooling phase, external restraints induce tensile stress at the slab thickness:

(21) $$\sigma_{top,tension}^{cp-II}=R\alpha_{T}\Delta T_{4}E_{cm,eff}$$

(22) $$\sigma_{center,tension}^{cp-II}=R\alpha_{T}\Delta T_{5}E_{cm,eff}$$

Special attention should be paid to the temperature differences $\Delta T_{1}$, $\Delta T_{2},\;\;\Delta T_{3}$, $\Delta T_{4},$ and $\Delta T_{5}$ depicted in the above equations. Equations (23)–(29) describe their calculations. For the self-balanced stress, the crucial is the maximum difference in temperature between the center and the top surface of the slab:

(23) $$\Delta T_{1}=T_{center}-T_{top}$$

Generally, in the case of restrained thermal stresses, the following values should be considered:

(24) $$\mathrm{temperature}\;\mathrm{rise}\;\mathrm{in}\;\mathrm{the}\;\mathrm{heating}\;\mathrm{phase}:\;\Delta T=T_{max}-T_{initial}$$

(25) $$\mathrm{temperature}\;\mathrm{drop}\;\mathrm{in}\;\mathrm{the}\;\mathrm{cooling}\;\mathrm{phase}:\;\Delta T=T_{max}-T_{final}$$

Therefore, due to the nonlinear temperature distribution at the slab thickness, these values should be computed for the center and the top of the slab, based on:

(26) $$\Delta T_{2}=T_{top}-T_{initial}$$

(27) $$\Delta T_{3}=T_{center}-T_{initial}$$

(28) $$\Delta T_{4}=T_{top}-T_{final}$$

(29) $$\Delta T_{5}=T_{center}-T_{final}$$

The total stress is a sum of the self-balanced and externally induced stresses. Therefore, Equations (30) and (31) can be written for the heating phase:

(30) $$\sigma_{top}^{hp-final}=\sigma_{top,tension}^{hp-I}+\sigma_{top,compression}^{hp-II}$$

(31) $$\sigma_{center}^{hp-final}=\sigma_{center,compression}^{hp-I}+\sigma_{center,compression}^{hp-II}$$

Similarly, the final stresses can be calculated for the cooling phase based on Equations (32) and (33):

(32) $$\sigma_{top}^{cp-final}=\sigma_{top,compression}^{cp-I}+\sigma_{top,tension}^{cp-II}$$

(33) $$\sigma_{center}^{cp-final}=\sigma_{center,tension}^{cp-I}+\sigma_{center,tension.}^{cp-II}$$

The last equations stated for the final stresses indicate that the restraint stresses reduce tensile self-balanced stresses in the heating phase (Equation (30)). In the cooling phase, external restraints enlarge tensile stresses inside the slab (Equation (33)). Moreover, the above equations precisely identify the area in the tension for which the reinforcement should be calculated. In the slab with the dominant internal restraints, a significant tension occurs at the top of the slab cross-section in the heating phase. In the case of a high level of external restraints, the tension is located in the interior of the slab and this area is prone to cracking. Therefore, the crack mechanism presented in Figure 3 has been theoretically confirmed.

Finally, it should be mentioned that a primary problem in the described procedure is the determination of the temperature values. It is necessary to determine not only the maximum temperature of the self-heating based on the heat released during the cement hydration but also the in-time development of the temperature at the center and the top of the slab. Among different methods, the iterative procedure provided by CIRIA C766 [35] can be useful for this purpose.

## 5. Comparative Study of an Exemplary Slab

An exemplary foundation slab with a thickness of 3 m and the dimensions 30 m × 30 m in a plan view is considered. It is assumed that the slab has been designed for mechanical loading and the evaluation of the reinforcement area to control early-age crack widths is made for the output from the structural project. The basic data are listed in Table 5. The assumed value of the limit crack width is 0.3 mm.

The main aim of the study is to compare different methods for reinforcement calculation. For this purpose, the simplified method and more precise approach based on CIRIA C766 [35] were compared. Additionally, two types of restraints are assumed in the exemplary slab. In the first case, the slip layer between the slab and lean concrete is considered. Therefore, only self-induced strains caused by internal restraints are considered. In the second case, it is assumed that the considered slab has been cast on the concrete base without any slip layer. Therefore, the restrained strains induced by the external restraints are considered.

### 5.1. Results from the Simplified Approach

In the simplified method, the actual level of induced thermal stresses and their distribution are ignored. The crack width is limited by using the suggested bar size. This involves introducing the reduced value of the maximum stress permitted in the reinforcement based on Table 1. The results of the calculation are depicted in Table 6 and Table 7. The required minimal reinforcement, $A_{s,min}$, for possible early-age cracking of thermal origin was calculated using the CIRIA C766 [35] recommendations (Table 2 and Table 3). The results indicate that the existing near-surface reinforcement of the slab is too small for early thermal cracks. The fulfillment of the assumed maximum crack width equal to 0.3 mm can be achieved by reducing the spacing of the bars to 9 cm ($A_{s}$ = 22.33 cm^2^) in the slab with the slip layer. Considering the externally restrained slab, an extremely high area of required reinforcement is obtained. In this case, the calculated near-surface reinforcement, reducing the maximum crack width to 0.3 mm, is ϕ25 at 7 cm.

Next, the recommendations from other standards have been compared. Particular discrepancies are visible for the slab with the slip layer, in which only the self-balanced stresses are considered. The following required reinforcement area was obtained:

- Based on Eurocode 2, with k = 0.65, $k_{c}$ = 1.0, and $A_{ct}\;$= 0.60 m^2^, the required $A_{s,min}$ is equal to 28.11 cm^2^. Thus, a greater area is obtained.
- Based on Eurocode 2, with k = 0.65, $k_{c}$ = 1.0 and considering the tensile zone covering only the surroundings of the reinforcement (Equation (10)), $A_{ct}$ = 0.17 m^2^), the required $A_{s,min}$ is equal to 7.96 cm^2^. Thus, a much lower area is obtained.
- Based on DIN EN 1992-1-1 / NA, with k = 0.52, $k_{c}$ = 1.0, and $A_{ct}$ = 0.60 m^2^, the required $A_{s,min}$ is equal to 22.49 cm^2^. Thus, a similar area is obtained.
- Based on DIN EN 1992-1-1 / NA, with $h_{sk}$ = 0.34 m (Equation (7)) and $A_{ct}\;$= 0.34 m^2^, the required $A_{s,min}$ is equal to 24.51 cm^2^. Thus, a greater area is obtained.

### 5.2. Results from the Approach Based on CIRIA C766

**Figure 5:**
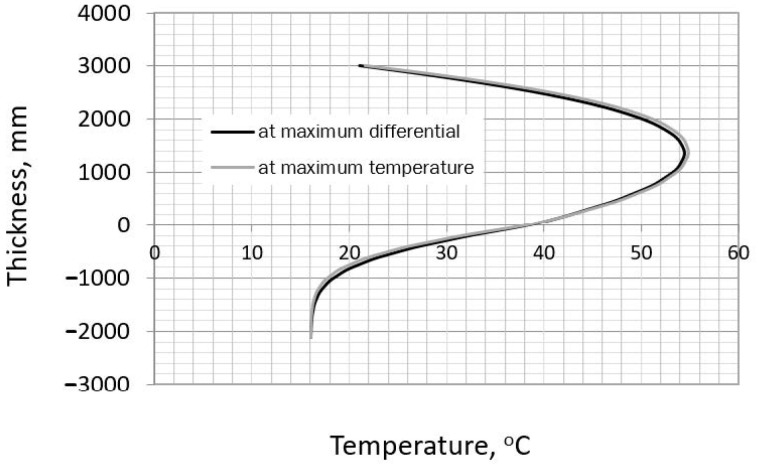
The temperature profile at the slab cross−section.

In this method, the actual thermal strains in the heating phase are considered. The temperature caused by the cement hydration can be estimated using the iterative method provided by CIRIA C766 [35]. The obtained values are listed in Table 8, while the temperature profile at the slab cross-section is presented in Figure 5.

Considering the slab with the slip layer, the results listed in Table 9 indicate that the existing near-surface reinforcement of the slab is sufficient to limit the early thermal cracks. The calculated crack width is equal to 0.08 mm and is much lower than the assumed limit of 0.3 mm. Nevertheless, it should be remembered that the presented calculation was performed for particular technological and material data. To keep the crack width within the acceptable limit, these assumptions should be fulfilled during the construction of the slab. Each change in the initial data influences the area of reinforcement required. Thus, an additional calculation has been made assuming the extremely high value of the maximum center-top difference of 60 °C, which is rather theoretical. In this case, the obtained crack width is equal to 0.16 mm and is still below the allowed value of 0.3 mm. Even in this extreme case, there is no need to increase the reinforcement area. Furthermore, assuming the reinforcement area, calculated with a small tensile area, is equal to 7.96 cm^2^ (Section 5.1), the obtained crack width is equal to 0.14 mm.

For the externally restrained slab, similar results are obtained (Table 10). Considering the existing near-surface reinforcement, the calculated crack width is equal to 0.09 mm. However, this width of the crack has been calculated considering only the external restrained strains. since the guideline recommends such an approach.

## 6. Discussion

**Figure 6:**
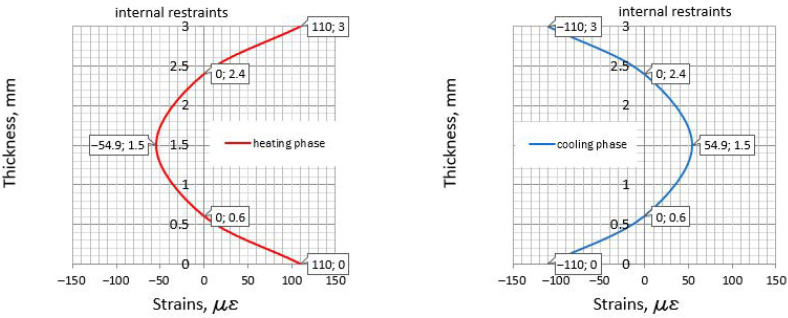
The strains in the slab with the slip layer.

**Figure 7:**
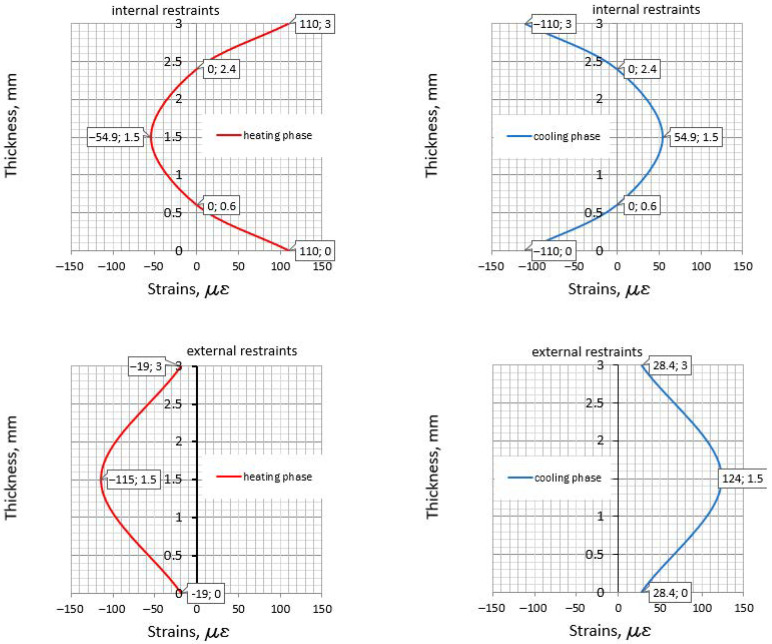
The strains in the externally restrained slab.

Finally, the results from Section 5 have been compared with the strain distribution determined based on the equations from Section 4. The required temperatures (Equations (15)–(22)) are listed in Table 11. The distribution of strains and their values are depicted in Figure 6 (slab with the slip layer) and Figure 7 (externally restrained slab).

Considering the slab with the slip layer, in which only the internal restraints exist, the following remarks based on all reviewed methods can be stated:

- At the top surface, in the heating phase, the induced tensile strain equal to 110 me has been obtained both based on CIRIA C766 and the precise analysis of the strains (Figure 6). This is obvious, since the same restraint factor, *R*, is assumed in both approaches.
- The center of the slab in the cooling phase is not considered in the method provided in CIRIA C766. The distribution presented in Figure 6 shows the value of the tensile strain induced in the cooling phase. It is relatively low and not exceeding the ultimate strain capacity. Thus, in the analyzed slab there is no need to analyze the cracking in the center area. Nevertheless, the tensile area exists and should be checked concerning the possible cracking.
- The results presented in Section 5.1 (simplified method) indicate serious discrepancies in the required area of reinforcement, ensuring the assumed limit value of the crack width. This is valid especially for the direct application of the Eurocode 2 standard, which does not provide detailed guidelines for reinforcement against early thermal effects in mass foundation slabs. The problem concerns mainly the unspecified recommendation for the tension area which should be used in the reinforcement calculations. Considering the slab with the slip layer and assuming the tension zone, $A_{ct}$, based on the actual stress distribution, a large reinforcement area is obtained to ensure an appropriate crack width.
- The results obtained from the CIRIA C766 method suggest that, for calculating the reinforcement in slabs with the internal restraints, the smaller area of the tension zone can be taken. Thus, instead of the actual area, $A_{ct}$, the effective area of concrete in tension, $A_{c,eff}$, around the reinforcement to a depth of $h_{c,eff}=2.5\left(c+\phi /2\right)$ may be applied.

The behavior of the externally restrained slab is more complex, since both the strains resulting from internal and external restraints should be analyzed (Figure 7). The main remarks from the comparative study can be written as follows:

- At the top surface, in the heating phase, the external restraints reduce the tensile strains to $\varepsilon_{r}=110-19=98\;\mu \varepsilon$. Nevertheless, for safety reasons, it is recommended to consider only the tensile strains related to the internal restraints.
- At the top surface, in the cooling phase, the external restraints reduce the compressive strains to $\varepsilon_{c}=-110+28.4=-81.6\;\mu \varepsilon$. This value is lower than the tensile strains induced in the heating phase. This seems to be an important observation, since a common belief that surface cracks close during the cooling phase may be incorrect.
- In the center of the slab, in the cooling phase, the maximum tensile strains are observed, equal to $\varepsilon_{r}=124+54.9=178.9\;\mu \varepsilon$. They are even greater than the tensile strains at the top surface, in the heating phase. These strains are also greater than the ultimate strain capacity, which is equal to $\varepsilon_{ctu}=66\;\mu \varepsilon$ (3-day concrete) and $\varepsilon_{ctu}=123\;\mu \varepsilon$ (28-day concrete). Thus, a crack in the center may be induced. Considering a more reasonable value for 28-day-old concrete (the end of the cooling phase) and the same reinforcement as applied at the top surface, the crack width is 0.11 mm. It seems that applying such reinforcement can be an effective method for reducing the crack developing from the center to the top surface.
- The simplified method (Section 5.1) applied for the externally restrained slab results in a huge reinforcement area (70.28 cm^2^). Following the simplified method, this reinforcement should be concentrated in the sub-surface area. In this case, the method based on CIRIA C766 also controls the crack width at the top surfaces and consider the subsurface reinforcement, but it takes the real strains. Nevertheless, both methods omit the share of the self-induced strains in the mass slab with additional external restraints.

## 7. Conclusions

The presented contribution investigated important engineering topics related to the determination of reinforcement withstanding early tensile strains in mass foundation slabs. This study provides an insight into the strain distributions and the relevant reinforcement for controlling early-age cracks of thermal origin. A comprehensive discussion and clarification of the guideline recommendations devoted to the reinforcement detailing are presented. A method for the identification of tensile strains in mass foundation slabs both in the heating and the cooling phase is also proposed. Next, the possible paths for calculating the reinforcement are presented through the example of mass foundation slabs with different levels of external restraints. 

First, it seems that the correct calculation of the reinforcement requires the identification of the tensile strain distribution in a mass slab. In this way, the areas of the slab where the reinforcement should be applied are identified. In particular, the difference in the behavior of the slab with internal restraints and the slab with additional external restraints is demonstrated. 

Next, the influence of the calculation method as well as the restraint conditions of the slab on the amount of reinforcement required were discussed. In this regard, the obtained results confirm the simplified method as very conservative, which may lead to an unnecessary increase in the slab’s subsurface reinforcement, especially in an externally restrained slab. The methods based on the analysis of tensile strains seem to be more accurate and economical approaches to avoid the unnecessary oversizing of the reinforcement. Finally, the analysis of strain distribution suggests applying the reinforcement in the interior of the externally restrained slab.

## Figures and Tables

**Table 1:** The maximum stress in reinforcement *σ_s_* dependent on bar diameter [33].

Steel Stress, MPa	Maximum Bar Size, mm		
	*w_k_* = 0.4 mm	*w_k_* = 0.3 mm	*w_k_* = 0.2 mm
160	40	32	25
200	32	25	16
240	20	16	12
280	16	12	8
320	12	10	6
360	10	8	5
400	8	6	4
450	6	5	–

**Table 2:** Tensile strength for concrete classes [34,35].

Concrete Class	C20/25	C25/30	C30/37	C35/45	C40/50	C45/55	C50/60	C55/67	C60/75
$f_{ctm}\left(3\;\mathrm{days}\right)$, MPa	1.32	1.53	1.73	1.92	2.12	2.27	2.44	2.52	2.61
$f_{ctm}$, MPa	2.21	2.56	2.90	3.21	3.51	3.80	4.07	4.21	4.35

**Table 3:** Recommendations for $A_{ct},k,\mathrm{and}\;k_{c}$ based on CIRIA C660 [34] and CIRIA C766 [35].

Value		Type of Induced Thermal Stress	
		Self-Induced (Internal Restraints)	Restrained Stress (External Restraints)
Coefficient	$k_{c}$	0.5	1.0 (pure tension)
Coefficient	k	1.0	1.0 for h < 300 mm 0.75 for h > 800 mm [31] 0.65 for h > 800 mm [32]
The thickness of the tensile area	−	0.2 h at each surface	h *
The area of the area in tension (for a length of 1 m of the slab)	$A_{ct}$	0.2 h·1 m	h·1 m

* h is the slab thickness.

**Table 4:** External restraint *R* for mass slabs [34,35].

Restraint Conditions	*R*
Massive pour cast onto blinding	0.1–0.2
Base of massive pour cast onto existing mass concrete	0.3–0.4

**Table 5:** Basic data for calculation.

Material and Technological Data	
Concrete class	C30/37
Cement type	CEM III (slag content is 58%)
Cement content	300 kg/m^3^
Water content	150 kg/m^3^
Aggregate type	gravel
Concrete density	2400 kg/m^3^
28-day compressive strength *f_cm_*	38 MPa
28-day tensile strength *f_ctm_*	2.9 MPa
Modulus of elasticity *E_cm_*	33 GPa
Reinforcement at the top and bottom surface in both directions	ϕ16 at 12 cm
Concrete cover	60 mm
Ambient temperature	15 °C
Initial concrete temperature	18 °C
Wind speed	4 m/s

**Table 6:** Results from the simplified approach—the slab with a slip layer.

Calculated Value	Source/Assumption	Value
$k_{c}$	Based on Table 3	0.5
k	Based on Table 3	1.0
$A_{ct},\;$ m^2^	$A_{ct}=0.2h\;\cdot 1\;m$, h=3 m	0.60
$\sigma_{s}$, MPa	Based on Table 1, ø16, $w_{k}=0.3\;\mathrm{mm}$	240
$f_{ct,eff}$, MPa	Based on Table 2	1.73
$A_{s,min}$, cm^2^	Equation (1)	21.63
$A_{s}$, cm^2^	Existing reinforcement, Table 5	16.75

**Table 7:** Results from the simplified approach—the slab with a high level of external restraints.

Calculated Value	Source/Assumption	Value
$k_{c}$	Based on Table 3	1.0
k	Based on Table 3	0.65
$A_{ct},$ m^2^	$A_{ct}=0.5h\;\cdot 1\;m$, h=3 m	1.5
$\sigma_{s}$, MPa	Based on Table 1, ø16, $w_{k}=0.3\;\mathrm{mm}$	240
$f_{ct,eff}$, MPa	Based on Table 2	1.73
$A_{s,min}$, cm^2^	Equation (1)	70.28
$A_{s}$, cm^2^	Existing reinforcement, Table 5	16.75

**Table 8:** The temperature based on the iterative method [35].

Calculated Value	Value
Maximum temperature in the center, °C	54.8
Maximum temperature at the top surface, °C	24.1
Maximum differential (center—top), $\Delta T_{}$, °C	33.5
Maximum temperature drop to the ambient temperature, °C	39.8

**Table 9:** Results from the approach based on CIRIA C766 [35]—the slab with a slip layer.

Calculated Value	Source/Vssumption	Value
ΔT, °C	Based on Table 7	33.5
$\alpha_{T}$, με /°C	Based on [34]	12
R	Recommended value	0.42
$K_{1}$	Recommended value	0.65
$\varepsilon_{r}$	Equation (12)	110
$\varepsilon_{ctu},\mu \varepsilon$	Based on [34]	66
Cracking risk	$\varepsilon_{r}>\varepsilon_{ctu}$	YES
$\varepsilon_{cr},\mu \varepsilon$	Equation (11)	76.7
$k_{1}$	recommended value	1.14
Bar diameter ϕ,m	Table 5	0.016
Bars spacing, m	Table 5	0.12
Bars cover, c,m	Table 5	0.06
$A_{s},{\mathrm{cm}}^{2}$	Existing reinforcement, Table 5	16.75
$A_{c,eff},m^{2}$	$A_{c,eff}=h_{c,eff}\times 1\;m$,	0.17
$\rho_{eff}$	$\rho_{eff}=A_{s}/A_{c,eff}$	0.00985
$s_{r,max},m$	Equation (9)	0.99
w,mm	Equation (9)	0.08

**Table 10:** Results from the approach based on CIRIA C766 [35]—the slab with external restraints.

Calculated Value	Source/Assumption	Value
ΔT, °C	Based on Table 7	39.8
$\alpha_{T}$, με/°C	Based on [34]	12
R	Recommended value	0.4
$K_{1}$	Recommended value	0.65
$\varepsilon_{r}$	Equation (12)	124
$\varepsilon_{ctu},\mu \varepsilon$	Based on [34]	66
Cracking risk	$\varepsilon_{r}>\varepsilon_{ctu}$	YES
$\varepsilon_{cr},\mu \varepsilon$	Equation (11)	91.2
$k_{1}$	Recommended value	1.14
Bar diameter ϕ,m	Table 5	0.016
Bars spacing, m	Table 5	0.12
Bars cover, c,m	Table 5	0.06
$A_{s},{\mathrm{cm}}^{2}$	Existing reinforcement, Table 5	16.75
$A_{c,eff},m^{2}$	$A_{c,eff}=h_{c,eff}\times 1\;m$,	0.17
$\rho_{eff}$	$\rho_{eff}=A_{s}/A_{c,eff}$	0.00985
$s_{r,max},m$	Equation (9)	0.99
w,mm	Equation (9)	0.09

**Table 11:** The temperature based on the iterative method [35].

Calculated Value	Value
Maximum differential (center-top), $\Delta T_{1}$, °C	33.5
Heating phase—temperature increase at the top surface, $\Delta T_{2},$ °C	6.1
Heating phase—temperature increase in the center, $\Delta T_{3},$ °C	36.8
Cooling phase—temperature drop at the top surface, $\Delta T_{4},$ °C	9.1
Cooling phase—temperature drop in the center, $\Delta T_{5},$ °C	39.8

## Data Availability

No new data were created or analyzed in this study. Data sharing is not applicable to this article.
